# A Pragmatic Pilot Cluster-Randomized Study of Tobacco Screening and Smoking Cessation Program for Community Pharmacies in Japan: FINE Program

**DOI:** 10.1155/2021/9983515

**Published:** 2021-12-03

**Authors:** Mitsuko Onda, Michiko Horiguchi, Masayuki Domichi, Naoki Sakane

**Affiliations:** ^1^Department of Social and Administrative Pharmacy, Osaka Medical and Pharmaceutical University, Osaka 569-1094, Japan; ^2^Cocokara Fine Healthcare Inc., Kansai branch, Osaka 542-0081, Japan; ^3^Division of Preventive Medicine, Clinical Research Institute, National Hospital Organization Kyoto Medical Center, Kyoto 612-8555, Japan

## Abstract

**Objectives:**

To investigate the effectiveness of a smoking cessation program (FINE program) in community pharmacies.

**Methods:**

A cluster-randomized controlled trial was conducted in 11 community pharmacies in Japan. The participants were randomly assigned to a pharmacist-led structured smoking cessation program (intervention group) or pharmacist-led usual care (control group). The intervention group was followed up over the telephone on the third day of smoking cessation, and ongoing follow-up and advice were provided according to the original smoking cessation guidebook developed for the current study based on a behavioral change approach. The control group received brief advice and ready-made pamphlets on smoking cessation from pharmacists upon their visit to these community pharmacies. The primary outcome was continued smoking cessation as determined by self-reporting and carbon monoxide monitoring with a microsmokerlyzer after 3 months.

**Results:**

Five hundred and seventy-two smokers who met the eligibility criteria visited the pharmacies included in the study. Of these individuals, 24 patients agreed to participate in the study. The quit rates were 45.5% and 18.2% in the intervention and control groups, respectively (*P* = 0.380, effect size = 0.60).

**Conclusion:**

Based on the effect size values, the FINE program may be effective to some extent, but the difference was not significant. We speculate that this is related to the small sample size due to difficulty in recruiting. Further studies with an effective recruitment method and larger sample sizes are needed to accurately verify the effectiveness of this program.

## 1. Introduction

Each year, more than 8 million people worldwide die from diseases linked to smoking tobacco products. As such, smoking is recognized as a serious health threat and classified as an epidemic by the World Health Organization [1]. Tobacco use is one of the most preventable causes of morbidity and mortality [2]. Since smoking increases the risk of morbidity and worsens lifestyle-related diseases, there is a need for innovative strategies to efficiently help people quit smoking [3]. In this context, we believe that pharmacies, as easily accessible social resources in the community, are the best place to provide smoking cessation support.

Pharmacies offer a wide range of smoking cessation aids and alternatives to smoking, and pharmacists can provide information and advice on their use [4]. Previous studies have shown that attending training programs on smoking cessation support enabled pharmacists to improve their counseling skills [5, 6] and increase smoking cessation rates [6–8]. Evidence also suggests that such training programs lead to behavioral changes in pharmacists that result in long-term persistence of provision of smoking cessation support [9] and result in savings in treatment costs [10].

A systematic review of the effectiveness of community pharmacists' support for smoking cessation included a meta-analysis of 10 research articles. It found that pharmacists' support for smoking cessation, either as a nonpharmacological intervention providing behavioral counseling and support alone or combined with pharmacological approaches such as nicotine replacement therapy, is potentially effective [3]. In a meta-analysis of seven research articles, community pharmacists provided weekly counseling compared to control groups that received only a few minutes of face-to-face support. The meta-analysis revealed that the support provided to the intervention groups was more effective compared to that provided to the control groups [4].

Separately, a meta-analysis of five research articles showed that a nonpharmacological pharmacist intervention providing behavioral counseling and support was somewhat effective in terms of smokers' self-reported quit rates and short- and long-term quit rates compared to those in the control group [11]. However, no clear benefit of nicotine replacement therapy was found [11]. A meta-analysis of 16 research articles revealed that the most common intervention method was the provision of individual counseling in an appointment-based manner [12]. However, all reviews indicated that accumulation of more evidence (studies in which intervention methods are clear and variability and bias are controlled as much as possible) is needed to draw more robust conclusions [3, 4, 11, 12].

In Japan, the Ministry of Health, Labour, and Welfare launched the “family pharmacy system” in 2016, which requires community pharmacies to be more deeply involved in the health management of patients as “health support pharmacies.” In addition, these pharmacies are required to undertake daily prescription audits, drug preparation, medication history management, consult with physicians, and offer medication guidance. In this system, support for smoking cessation is explicitly stated as a role to be played by pharmacists [13]. However, the effectiveness of smoking cessation support by community pharmacists in Japan has not been clearly elucidated. There is one report examining the supportive effect of teaching smoking cessation aids, but no clear results have been obtained [14].

To implement smoking cessation support in busy Japanese pharmacies in the future, two main issues need to be addressed: (1) the development of a practical and systematic training program for community pharmacists and (2) the establishment of a simple support method that can be implemented in a short period of time in between dispensing operations. Therefore, the authors developed and confirmed the feasibility of a training program on smoking cessation support for community pharmacists to ensure easy implementation of personalized smoking cessation support [15]. The purpose of this pilot study was to investigate the effectiveness of the smoking cessation support program.

## 2. Materials and Methods

### 2.1. Trial Design

This was a pragmatic cluster-randomized trial (registration number: UMIN000032063; May 1, 2018).

### 2.2. Participants

Community pharmacies were directly recruited via explanatory meetings, resulting in the participation of 11 pharmacies in Osaka Prefecture. Participants who could be included in the clusters were current smokers aged ≥20 years and who were expected to visit a community pharmacy during the 3-month study period. The participant exclusion criteria were as follows: intolerance to smoking cessation aids (due to an allergy or adverse reaction), major neurocognitive disorder under DSM-5, a temporomandibular joint disorder, or pregnancy.

### 2.3. Randomization

Pharmacies were considered to be clusters and divided into categories according to size (stratified by the number of prescriptions per month: small, 0-999; medium, 1,000-1,999; and large, more than 2,000). Pharmacies were randomized into either intervention group or control group in a 1 : 1 ratio, using a blocked randomization method (block size 4). The stratified random allocation sequence was generated using computer-generated random numbers by a biostatistician at the Kyoto Medical Center Clinical Research Center. Pharmacists in each collaborative pharmacy enrolled and assigned participants.

### 2.4. Recruitment

The pharmacists who participated in the study displayed posters and introduced the program to those visiting the pharmacies. They also asked those who visited the pharmacies to fill out a smoking status questionnaire, approached those who were interested in quitting smoking, explained the program verbally to them, and obtained written consent for participation in the current study. The recruitment period extended from April to June 2018, with the goal of enrolling four patients per cluster (pharmacy).

### 2.5. Blinding

The intervention was assumed to be behavioral, and the pharmacists were not blinded. However, the participants and analysts were blinded to group assignment.

### 2.6. Intervention Group

#### 2.6.1. Pharmacist Training

All participating pharmacists were required to complete training prior to study commencement [15]. An introductory e-learning course was provided by the Japan Smoking Cessation Training Outreach Project. Pharmacists who completed the e-learning course then participated in an additional 4-h training program developed by the investigators. The program introduced pharmacists to the FINE program that included determinations of a behavioral change stage, degree of smoking harm, introduction to activities conducted at smoking cessation clinics, instructions to patients on how to use nicotine patches, how to recruit participants by using a questionnaire, how to use a piCO smokerlyzer (Harada Industry), and evaluation criteria (participants were considered to have quit smoking if their score level was ≤6 ppm). Any questions were addressed by the investigators, who also discussed practical aspects of smoking cessation support. Finally, the pharmacists were trained by the investigators in communication skills for all of the above interpersonal tasks (see Table 1) [15].

#### 2.6.2. Intervention (FINE Program)

The pharmacists provided smoking cessation support to each participant as soon as they enrolled in the study, and the last participant completed a 3-month follow-up in September 2018. Eighteen pharmacists performed the interventions. The participants in the FINE program received an original guidebook that included a smoking diary, an explanation of how to use and record the start date of using a smoking cessation aid, and how to measure the CO concentration in exhaled air (this activity was voluntary). Then, on day 3, the pharmacists telephoned the participants asking if they had been smoking or had experienced nicotine withdrawal symptoms and followed up with encouragement, counseling, and advice at weeks 2, 4, 6, and 8, either in person or over the phone. Furthermore, the pharmacists determined whether the participant used their smoking diaries and continued to refrain from smoking. They also checked whether the participants were smoking and measured the CO concentration in their breath at 12 weeks (see Table 2).

### 2.7. Control Group

Seventeen participating pharmacists completed the same e-learning course as did the participating pharmacists catering to the intervention group. Pharmacists who completed the e-learning course were only taught how to use nicotine patches and the piCO smokerlyzer and how to recruit participants by using a questionnaire by the investigators. The pharmacists provided usual interventions, such as asking patients to confirm that they had quit smoking when they visited the pharmacy and giving them smoking cessation pamphlets prepared by a pharmaceutical company.

### 2.8. Outcomes

The primary outcome was the percentage of participants who had quit smoking at the end of the 3-month follow-up period (3-month quit rate).

### 2.9. Measurements

Confirmation of continuous smoking cessation was based on the participants' self-report and measurement of CO concentration in exhaled air (participants were considered to have quit smoking if their score level was ≤6 ppm).

### 2.10. Sample Size

Due to the wide range of results from previous studies on the effectiveness of smoking cessation support in community pharmacies, the effect size was set based on the work of [16]. Accordingly, the smoking cessation rate without professional help was 3-5%, while that after 6 months increased to 35%-55% when assistance of medical professionals was available. Therefore, since the difference was estimated to be 30-50%, an intermediate value of 40% was used as the tentative effect size. In addition, an alpha error of 5% and power of 80% were set. A two-tailed significance test was used to calculate the sample size required for each group. Using an intraclass correlation of 0.05, we set the sample size to 24 participants per arm.

### 2.11. Statistical Analysis

Continuous variables are presented as mean and standard deviation (SD) values, and categorical variables are presented as numbers and percentages. The difference in the primary outcome between the groups was compared using the *χ*^2^ test. Chi-square test adjustment for clusters (pharmacies) was performed using the clchi2 command in Stata. A *P* value of less than 0.05 was considered statistically significant, and the effect size was calculated. The analysis was performed using Stata 13 and *R*.

### 2.12. Ethics Approval

The study was approved by the ethics committee of Osaka University of Pharmaceutical Sciences (0054). All participants provided written informed consent for participation in the study before enrollment, which was in compliance with the principles of the Declaration of Helsinki. Furthermore, the Consolidated Standards of Reporting Trials statement was followed for the reporting of the study [17].

## 3. Results

**Figure 1:**
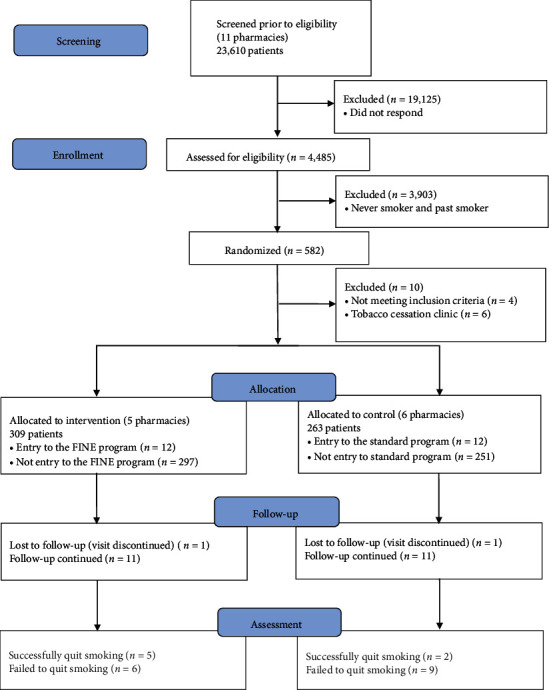
Recruitment, allocation, and 3-month follow-up of study subjects.

Eleven pharmacies participated in the recruitment, which began on April 15, 2018; patient assignment is shown in Figure 1. Of 4,485 people who answered the survey during the registration period, 582 were smokers (13.0%). Five pharmacy clusters assigned to the intervention group, and six pharmacy clusters assigned to the control group surveyed 309 and 263 participants, respectively. The enrolment rate was 4.2% (24/572), resulting in a total of 12 smokers in the intervention group of five clusters (two or three patients per cluster) and 12 smokers in the control group of six clusters (two patients per cluster). Intergroup differences in sex, age, smoking status (type and quantity), and interest in quitting were not significant. However, it was suggested that the intervention group had a higher percentage of prescriptions for diabetes and dyslipidemia medications and inhalers (See Table 3).

The continuous smoking cessation rates at 3 months were 45.5% (5/11) and 18.2% (2/11) in the intervention and control groups, respectively (*P* = 0.380, effect size = 0.60). Regarding the effect size, the values of 0.1, 0.3, and 0.5 indicated small, medium, and large effects, respectively [18]. The exhaled CO concentration scores (ppm) of the five successful quitters in the intervention group after 12 weeks were 1, 2, 0, 4, and 0, respectively. The exhaled CO concentration scores of the two successful quitters in the control group after 12 weeks were 5 and 0, respectively.

One patient in the intervention group was lost to follow-up: he stopped coming to the pharmacy during the study period and could not be followed up by phone. However, he had quit smoking after developing pneumonia at the time of his subsequent visit. He commented that it was his participation in the FINE program that motivated him to quit smoking. One patient in the control group was also lost to follow-up: he stopped coming to the pharmacy due to moving. Therefore, his subsequent progress is unknown. These two individuals were excluded from the calculation of the smoking cessation rate.

## 4. Discussion

To our knowledge, this is the first study in Japan to develop a support protocol for individual smoking cessation and training program for community pharmacists that could be implemented in 3 to 5 min during regular medication guidance. Furthermore, the effectiveness of the intervention was assessed in a randomized controlled trial. The follow-up period in this study was 3 months, which is the follow-up period for smoking cessation support specified in the public health guidance by physicians and public health nurses in Japan.

The difference in the smoking cessation rate between the two groups was 27.3%. In previous studies on smoking cessation, support by community pharmacists conducted overseas, apart from daily work, frequent counseling for 15 to 60 min during the first session and 10 to 30 min during the second and subsequent follow-ups, counseling at locations where walk-ins are available, and collaboration with nurse practitioners, computer-assisted programs, and telephone follow-up have been shown to make a significant difference in smoking cessation rates, ranging from 9.9 to 77.0% [19–22]. Compared to these results, our study suggests that the FINE program may enable community pharmacists to efficiently provide advice to smokers who have decided to quit. The results also suggest that the FINE program could play an important role in the application of motivational interviewing to support smoking cessation in patients affected by chronic diseases such as diabetes, dyslipidemia, and respiratory diseases.

However, there are limitations to the current study. First, we were unable to reliably test our hypotheses due to a low recruitment rate and an insufficient number of participants. This clearly shows that it is difficult to recruit patients to smoking cessation programs in community pharmacies under the current situation in Japan. Specifically, we found that the reasons for the small sample size were that the expected effect size was too large, and the number of pharmacies was insufficient due to a high estimate of the expected number of patients that each pharmacy could recruit. Second, because we did not specify “interest in smoking cessation” in the patient selection criteria, patients with low interest in smoking cessation might have dropped out directly. Therefore, in the future, we will need to reexamine our recruitment methods, understand the level of interest in smoking cessation in advance, and train pharmacists to respond accordingly, as well as reduce the number of recruits per pharmacy and increase the number of pharmacies. Third, the study was conducted unblinded because it would be difficult to blind behavioral interactions between patients and pharmacists.

The issues pertaining to recruitment methods in Japanese community pharmacies that were clarified in the current study need to be further considered from the perspectives of both patients and pharmacists. According to a survey on effective ways to recruit smokers and reasons for smokers who are not interested in quitting to participate in smoking cessation support research, common recruitment methods include word of mouth, posters, and referrals to outpatient clinics for smoking cessation. Further, the most common reasons for participating in such research were, in order of frequency, financial incentives (44.7%), learning about research and smoking cessation support (43%), learning about smoking and risks (40%), and future smoking cessation support (23.9%). Therefore, to increase the recruitment rate in the future, we believe that it would be useful to add more specific information about the content of the smoking cessation support program and the benefits of smoking cessation to the explanatory document in an easy-to-understand manner before obtaining consent [23].

Many pharmacies have not incorporated smoking cessation support into their daily operations is that it takes time and effort to recruit patients under tight time constraints [24]. Additionally, community pharmacists may feel they lack the counseling skills needed to help smokers quit [25]. For community pharmacies to proactively reach out to smokers, a simplified approach to smoking cessation support is needed, and its effectiveness needs to be objectively evaluated [25]. A study among Canadian pharmacists found that smoking cessation training in undergraduate and postgraduate education was associated with an increased belief that the role of a pharmacist includes providing effective counseling for smoking cessation. Therefore, the training program developed may need to be modified further to address this issue [26].

## 5. Conclusions

Based on the effect size values, the FINE program may be effective to some extent; however, the difference was not significant. We speculate that this is related to the small sample size due to the low recruitment rate. Therefore, further studies with an effective recruitment method and larger sample sizes are needed to accurately verify the effectiveness of this program.

## Figures and Tables

**Table 1:** Training program and follow-up during the study (citation from reference [15]).

Training before the study	Method
Self-learning before smoking support training (J-STOP) (180-240 min)	e-learning
The approach to smoking cessation in clinical practice	Text/video
(i) Harmful effects of smoking	
(ii) Effects of smoking cessation	
(iii) Nicotine dependence	
(iv) Pharmacotherapy for smoking cessation	
Case series	Video
(i) Male in the precontemplation stage of change for smoking cessation	
(ii) Male in the contemplation stage of change for smoking cessation	
(iii) Female in the contemplation stage of change for smoking cessation	
Q&A practice	

Theory (120 min)	
How to determine the behavioral change stage	Lecture
Smoking cessation clinics provided by doctors	Lecture
How to explain the harms of smoking	Discussion
How to use the smokerlyzer	Practice
Explanation of measurement results	Practice
How to use the questionnaire	Discussion
Preparation for smoking cessation	Discussion
How to use and select smoking cessation aids	Discussion

Practice (120 min)	
Explanation of the FINE program	Lecture
How to use materials for smoking cessation support	Discussion
How to recruit participants	Discussion
Recruitment using a questionnaire	Role-playing
Practice providing smoking cessation support using the FINE program	Role-playing

**Table 2:** The flow of the FINE program (citation from reference [15]).

Timing	Action
The first visit	Set of the start date of smoking cessation
	Measurement of CO concentration in exhaled air (voluntary)
	Delivery of the guidebook
	Explanation of how to use the guidebook
	Selection of the course that would be used to quit smoking
	The introduction of a smoking cessation aid (if necessary)

Day 3	Check whether or not participants had been smoking
Follow-up on the telephone	Check for nicotine withdrawal symptoms
	Words of encouragement
	Check for side effects of the smoking cessation aid (if necessary)

Weeks 2, 4, 6, and 8	Check whether or not participants had been smoking
Follow-up at the pharmacy or over the telephone	Check whether or not participants had been filling in their smoking diaries and give advice
	Words of encouragement
	Check for side effects of the smoking cessation aid (if necessary)

Assessment	Check whether or not participants had been smoking
	Measurement of CO concentration in breath at week 12

**Table 3:** Backgrounds of smoking patients.

Parameter	Intervention group (FINE program participants) (*n* = 12)	Control group (usual program participants) (*n* = 12)	*P*
Mean (SD) age	63.8 (8.17)	58.3 (21.73)	0.426
Male (%)	83.3	50.0	0.193
Antihypertensive drugs (yes: %)	50.0	33.3	0.168
Drugs for treating dyslipidemia	75.0	8.3	0.002
Drugs for diabetes	33.3	8.3	0.029
Inhaled drugs	16.7	8.3	0.018
Smoking status (%)			
Traditional cigarettes	91.7	75.0	0.122
Heated-not-burn/electronic tobacco products	0.0	25.0	
Both	8.3	0.0	
Mean (SD) amount consumed per day			
Traditional cigarettes	14.8 (7.08)	12.4 (5.90)	0.423
Heated-not-burn/electronic tobacco products	15.0 (0.00)	9.7 (8.51)	0.642
Mean (SD) duration of smoking in years	36.3 (14.5)	32.4 (22.3)	0.630
Stage of change (%)			
Indifference	0.0	0.0	0.504
Precontemplation	33.3	8.3	
Contemplation	25.0	33.3	
Preparation	33.3	50.0	
Unknown	8.3	8.3	

## Data Availability

The datasets generated during and/or analyzed during the current study are available from the corresponding author on reasonable request.
